# A Tensor-Based Subspace Approach for Bistatic MIMO Radar in Spatial Colored Noise

**DOI:** 10.3390/s140303897

**Published:** 2014-02-25

**Authors:** Xianpeng Wang, Wei Wang, Xin Li, Junxiang Wang

**Affiliations:** College of Automation, Harbin Engineering University, No. 145 Nantong Street, Harbin 150001, China; E-Mails: wangxianpeng@hrbeu.edu.cn (x.W.); xinxin-forever@sohu.com (x.L.); wjxiang2013@126.com (J.W.)

**Keywords:** MIMO radar, DOD and DOA estimation, spatial colored noise, higher-order singular value decomposition

## Abstract

In this paper, a new tensor-based subspace approach is proposed to estimate the direction of departure (DOD) and the direction of arrival (DOA) for bistatic multiple-input multiple-output (MIMO) radar in the presence of spatial colored noise. Firstly, the received signals can be packed into a third-order measurement tensor by exploiting the inherent structure of the matched filter. Then, the measurement tensor can be divided into two sub-tensors, and a cross-covariance tensor is formulated to eliminate the spatial colored noise. Finally, the signal subspace is constructed by utilizing the higher-order singular value decomposition (HOSVD) of the cross-covariance tensor, and the DOD and DOA can be obtained through the estimation of signal parameters via rotational invariance technique (ESPRIT) algorithm, which are paired automatically. Since the multidimensional inherent structure and the cross-covariance tensor technique are used, the proposed method provides better angle estimation performance than Chen's method, the ESPRIT algorithm and the multi-SVD method. Simulation results confirm the effectiveness and the advantage of the proposed method.

## 1. Introduction

Recently, multiple-input multiple-output (MIMO) radar [1–3] has drawn increasing attention and has become a hot research topic in the area of radar. MIMO radar uses multiple antennas to emit simultaneously orthogonal waveforms instead of the coherent waveforms, which are used in the phased-array radar, and this waveform diversity endows MIMO radar with superior performance relative to phased-array radar. Based on the configuration of transmit and receive antennas, MIMO radar can be grouped into two classes. One is called statistical MIMO radar [2], which can solve the problem of target scintillation, due to the widely spaced transmit/receive antennas. The other is called colocated MIMO radar [3], including bistatic and monostatic MIMO radar [4,5], whose transmit antennas and receive antennas are close spaced. The colocated MIMO radar can obtain the virtual aperture, which is larger than the real aperture, so it brings a lot of advantages, such as narrower beamwidth and lower sidelobes, higher angular resolution and angular estimation accuracy.

Angle estimation is an important aspect in array signal processing and MIMO radar [6–14]. In bistatic MIMO radar, the direction of departure (DOD) and the direction of arrival (DOA) need to be estimated simultaneously. In [8], a two-dimensional Capon estimator is applied to estimate DOD and DOA, which are paired automatically. However, it has a heavy computational burden owing to the two-dimensional spectrum searching. In order to alleviate the computational burden, the estimation of signal parameters via rotational invariance technique (ESPRIT) [9,10] is employed to DOD and DOA estimation. The rotational invariance properties of both the transmit and receive arrays are investigated in [9], then the DOD and DOA are determined through two independent 1D ESPRITs. However, an additional pairing operation is required. In [10], the relationship between two 1D ESPRIT is investigated. In [11], the real-valued ESPRIT (unitary ESPRIT) is proposed to estimate DOD and DOA. It has lower computational complexity and slightly better angle estimation performance compared with ESPRIT [9,10]. A multi-singular value decomposition (multi-SVD) method is presented for DOD and DOA estimation in [12]. It provides better angle estimation than the traditional eigenvalue decomposition (EVD)/SVD method. The above schemes can only be used for angle estimation in the presence of spatial Gaussian white noise. In [13], an ESPRIT-based method for bistatic MIMO radar DOD and DOA estimation is proposed, which can eliminate spatial colored noise. However, it is only effective for three transmit antennas configuration. By dividing the transmit array into two subarrays, a combined ESPRIT and SVD of the cross-correlation matrix method (denoted as Chen's method) is presented in [14], which is effective for MIMO radar with three or more transmit antennas to eliminate the influence of spatial colored noise.

However, in the subspace methods [13,14], the received signals are stacked into a special structure matrix, ignoring the multidimensional structure inherent in the received signals after matched filters. In this paper, a tensor-based frame is considered for the received signals, which exploits the multidimensional inherent structure and a novel tensor-based subspace for bistatic MIMO radar in the presence of spatial colored noise is proposed. Firstly, utilizing the multidimensional structure inherent in the received signals after matched filters, the received signals can be packed into a third-order measurement tensor. Then, the measurement tensor is divided into two sub-tensors, and a cross-covariance tensor is formulated to eliminate the spatial colored noise by exploiting the orthogonal characteristic of matched filters. Finally, the higher-order singular value decomposition (HOSVD) technique is employed to formulate the signal subspace. The DOD and DOA are estimated through the ESPRIT algorithm, which are paired automatically. Theoretical analysis and simulation results validate that the proposed method suppresses spatial colored noise more efficiently and provides better angle estimation performance than Chen's method, the ESPRIT algorithm and the multi-SVD method, especially at the low signal-to-noise ratio (SNR) region.

The rest of the paper is organized as follows. The tensor basics and signal model are presented in Section 2. A tensor-based subspace approach for angle estimation in the presence of spatial colored noise is proposed in Section 3. The computational complexity of the method is evaluated in Section 4. In Section 5, simulation results are provided to verify the performance of the proposed algorithm. Finally, Section 6 concludes this paper.

*Notation*:Scalars, column vectors, matrices and tensor are expressed by regular, bold lowercase, bold uppercase and bold calligraphic letters, respectively. [**A**]_*i*,*j*_ and 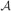
_*i*,*j*,*k*_ stand for the (*i*,*j*) and (*i*,*j*, *k*) element of a matrix, **A**, and a tensor, 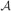. (·)^H^, (·)^T^, (·))^−1^ and (·)^*^ denote the Hermitian transpose, transpose, inverse and complex conjugation without transposition, respectively. ⊗ and ⊙ denote the Kronecker operator and the Khatri-Rao product, respectively. diag(·) denotes the diagonalization operation, and arg(*γ*) denotes the phase of *γ*.

## 2. Tensor Basics and Signal Model

### 2.1. Tensor Basics

For the readers' convenience, several tensor operations are introduced firstly, which refer to [15,16].

**Definition 1** (Matrix Unfolding):The three standard unfoldings of a third-order tensor, 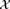 ∈ ℂ*^I^*^×^*^J^*^×^*^K^*, denoted by [ 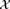]_(1)_ ∈ ℂ*^I^*^×^*^JK^* [ 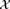]_(2)_ ∈ ℂ*^J^*^×^*^IK^* and [ 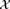]_(3)_ ∈ ℂ*^K^*^×^*^IJ^*, can be expressed as [[ 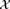]_(1)_]*_i_*,_(_*_k_*_−1)_*_J_*_+_*_j_* = [ 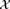]_*i*,*j*,*k*_, [[ 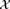]_(2)_]*_j_*,_(_*_i_*_−1)_*_K_*_+_*_k_* = [ 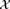]_*i*,*j*,*k*_ and [[ 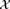]_(3)_]*_k_*,_(_*_j_*_−1)_*_I_*_+_ *_i_* = [ 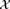]_*i*,*j*,*k*_, respectively.
**Definition 2** (Mode*-n* Tensor-Matrix Product):The mode*-n* product of 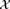 ∈ ℂ^*I*_1_^^×^^*I*_2_^^×⋯×^^*I*_N_^ by a matrix, **A** ∈ ℂ*^J_n_^*^×^*^I_n_^*, is denoted by 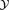 = 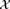 ×*_n_* **A**, where 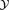 ∈ ℂ^*I*_1_^^×^^*I*_2_^^×⋯×^*^I_n_^*_−1_^×^*^J_n_^*^×^*^I_n_^*_+1_^×⋯×^*^I_N_^* and ${\left[Y\right]}_{i_{1},i_{2},\cdots ,i_{n-1},j_{n},j_{n+1},\cdots ,i_{N}}=\sum_{i_{n}=1}^{I_{n}}{\left[X\right]}_{i_{1},i_{2},\cdots ,i_{n-1},i_{n},i_{n+1},\cdots ,i_{N\cdot [A]j_{n},i_{n}}}$
**Definition 3** (The Properties of the Mode Product):The properties of the mode product are shown as follows:
  (1) $$\begin{array}{l} \begin{array}{rr} X\times_{n}\text{A}\times_{m}\text{B}=X\times_{m}\text{B}\times_{n}A, & m\neq n\\ X\times_{n}\text{A}\times_{n}\text{B}=X\times_{n}(\text{BA}) \end{array} \end{array}$$
  (2) $$\begin{array}{c} {\left[X\times_{1}{\text{A}}_{1}\times_{2}{\text{A}}_{2}\times \cdots \times_{K}{\text{A}}_{K}\right]}_{\left(n\right)}\\ ={\text{A}}_{n\cdot }{\left[X\right]}_{(n)\cdot }{\left[{\text{A}}_{n+1}⊗{\text{A}}_{n+2\cdots }⊗{\text{A}}_{K}⊗{\text{A}}_{1}\cdots ⊗{\text{A}}_{n-1}\right]}^{\text{T}} \end{array}$$

### 2.2. Bistatic MIMO Radar Signal Model

**Figure 1.**
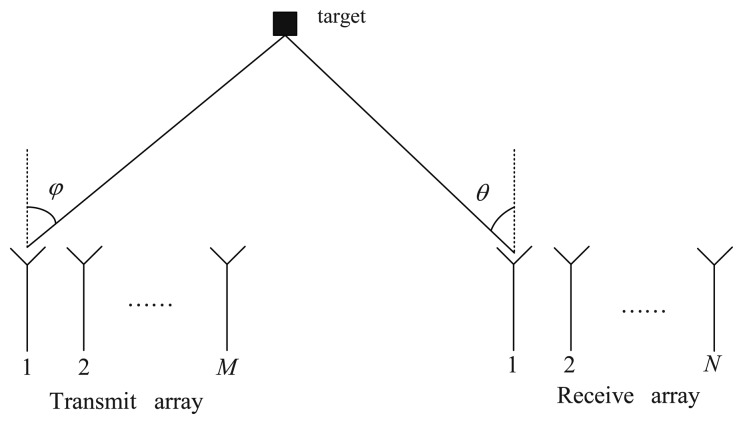
Bistatic multiple-input multiple-output (MIMO) radar scenario.

Consider a narrowband bistatic MIMO radar system with *M* colocated antennas for the transmit array and *N* colocated antennas for the receive array, shown in Figure 1.

Both the transmit array and receive array are uniform linear arrays (UALs), and the inter-element spaces of the transmit and receive arrays are half-wavelength. At the transmit array, the transmit antennas emit the orthogonal waveforms **S** = [**s**_1_, **s**_2_, ⋯, **s***_M_*]^T^ ∈ ℂ*^M^*^×^*^K^*, where *K* is the number of samples per pulse period. All the targets are modeled as a point-scatterer in the far-field, and it is assumed that there are *P* uncorrelated targets in the same range-bin of interest. ${\left\{\varphi_{p}\right\}}_{p=1}^{P}$ and ${\left\{\theta_{p}\right\}}_{p=1}^{P}$ are the DOD and DOA with respect to the transmit and receive array normal, respectively. We consider a coherent processing interval (CPI) consisting of *L* pulses, then the baseband received signal for the *l*-th pulse period at the output of the receive array can be written as [3,14]:

(3) $$\begin{array}{cc} X_{l}=B\Sigma_{l}AS+W_{l}, & l=1,2,\ldots ,L \end{array}$$

where **B** = [**b**(*θ*_1_),⋯ , **b**(*θ_P_*)] ∈ ℂ*^N^*^×^*^P^* and **A** = [**a**(*φ*_1_), ⋯ , **a**(*φ_P_*)] ∈ ℂ*^M^*^×^*^P^* are the receive steering and the transmit steering matrix, respectively; **b**(*θ_p_*) = [1, *e^jπ^* ^sin^ *^θ_p_^*, ⋯ , *e^jπ^*^(^*^N^*^−1) sin^ *^θ_p_^*]^T^ ∈ ℂ*^N^*^×1^ and **a**(*φ_p_*) = [1, *e^jπ^* ^sin^ *^φ_p_^*, ⋯, *e^jπ^* ^(^*^M^*^−1)sin^ *^φ_p_^*]^T^ ∈ ℂ*^m^*^×1^ are the receive steering vector and transmit steering vector of the *p*-th target, respectively. Σ*_l_* = daig(c*_l_*) with c*_l_* = [*β*_1_*e^j^*^2^*^πf^_d_*_1_*^lT_r_^*, ⋯,*β_P_ e^j^*^2^*^πf^_dp_^lT_r_^*], *f_dP_* is the Doppler shift of the *p*-th target and *T_r_* is the pulse repetition interval and ${\left\{\beta_{p}\right\}}_{p=1}^{P}$ are the reflection coefficients. **W***_l_* ∈ ℂ*^N^*^×^*^K^* is the noise matrix, and the columns of **W***_l_* are independent and identical distribution complex Gaussian random vectors with zero mean and an unknown covariance matrix, **Q̂**. Unlike traditional phased-array radar, MIMO radar transmits mutually orthogonal waveforms, i.e., $\left(1/K)s_{m}s_{m}^{\text{H}}=1,s_{i}s_{j}^{\text{H}}=0(i,j=1,2,\ldots M,i\neq j\right)$. Then, the received signals are matched by *M* transmitted waveforms, respectively. For the *l*-th pulse period, the output of the matched filter with the *m*-th transmitted waveform can be expressed as:

(4) $$\begin{array}{cc} Y_{l,m}=BD_{m}c_{l}^{\text{T}}+N_{l,m}, & l=1,2,\ldots ,L \end{array}$$

where $Y_{l,m}=(1/L)X_{l}s_{m}^{\text{H}}\in {\text{C}}^{N\times 1}$, **D***_m_* = diag([*a_m_* (*φ*_1_),…, *a_m_*(*φ_P_*)]), where *a_m_*(*φ_p_*) is the *m*-th element of **a**(*φ_p_*). $N_{l,m}=(1/L)W_{l}s_{m}^{\text{H}}\in {\text{C}}^{N\times 1}$ is the noise vector after the matched filter with the *m*-th transmitted waveform, which is an independent, zero-mean complex Gaussian distribution with an unknown covariance matrix, **Q̂,** and satisfied with $\text{E}[N_{l,i},N_{l,j}^{H}]=0(i,j=1,2,\ldots M,i\neq j)$.

## 3. Tensor-Based Subspace Approach for Angle Estimation

According to Equation (4), the received signals are matched with all the transmitted waveforms. Then, we have:

(5) $$\begin{array}{cc} Y_{l}=[Y_{l,1},\ldots ,Y_{l,M}]=B\Sigma_{l}A+N_{l}, & l=1,2,\ldots ,L \end{array}$$

In the conventional subspace-based methods, the received signals in Equation (5) are packed into a special structure matrix as **Y** = [vec(**Y**_1_)), vec(**Y**_2_),…, vec(**Y***_L_*)], which ignores the multidimensional structure inherent in the received signals. Based on the concept of **Definition 1**, it can be seen that the received signals for each pulse is a slice of a third-order tensor along the pulse direction. Therefore, by collecting *L* pulses, a third-order measurement tensor, 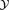, is constructed, which is satisfied with ${\left[Y\right]}_{\left(3\right)}^{\text{T}}=Y$. According to $\text{E}[N_{l,i},N_{l,j}^{H}]=0(i,j=1,2,\ldots M,i\neq j)$, it is implied that the output of the colored noise has the orthogonal characteristic between different matched filters. In order to exploit this characteristic, the measurement tensor, 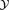, is divided into two sub-tensors, which is shown as:

(6a) $$Y_{1}=Y\times_{2}F_{1}$$

(6b) $$Y_{2}=Y\times_{2}F_{2}$$

where **F**_1_ = [**I**_*M*_1__,**0**_*M*_1___×(_*_M_*_−__*M*_1___)_], **F**_2_ = [**0**_*M*_2×(___*M*_−___*M*_2)__,**I**_*M*_2__] with *M*_1_ + *M*_2_ = *M*. According to Equation (6), we have:

(7a) $$\begin{array}{cc} {\left[Y_{1}\right]}_{:,:,l}=[Y_{l,1},\ldots ,Y_{l,M_{1}}]=B\Sigma_{l}A_{1}+N_{l}^{1}, & l=1,2,\ldots ,L \end{array}$$

(7b) $$\begin{array}{cc} {\left[Y_{2}\right]}_{:,:,l}=[Y_{l,M_{1}+1},\ldots ,Y_{l,M}]=B\Sigma_{l}A_{2}+N_{l}^{2}, & l=1,2,\ldots ,L \end{array}$$

where **A**_1_ = **F**_1_**A** and **A**_2_ = **F**_2_**A**. According to Equation (7), it is indicated that the measurement tensor data, 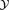
_1_ and 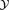
_2_, are obtained from different matched filters, i.e, 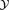
_1_ is the output of the first *M*_1_ matched filters and 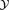
_2_ is the output of the residual *M*_2_ = *M* − *M*_1_ matched filters. Thus, we have $\text{E}[{\left(N_{l}^{1}\right)}^{\text{H}},N_{l}^{2}]=0(l=1,2,\ldots ,L)$ owing to $\text{E}[N_{l,i},N_{l,j}^{\text{H}}]=0(i,j=1,2,\ldots M,i\neq j)$. Then, a fourth-order cross-covariance tensor, 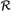
_21_ ∈ ℂ*^N^*^×^*^M^*_2_^×^*^N^*^×^*^M^*_1_, is formulated as:

(8) $$R_{21}=\frac{1}{L}Y_{2}•Y_{1}^{*}$$

where ${\left[R_{21}\right]}_{n,q,i,j}=1/L\sum_{l=1}^{L}{\left[Y_{2}\right]}_{n,q,l}{\left[Y_{1}\right]}_{i,j,l}^{*}$, *n*, *i* = 1,…, *N*.*q* = 1,…,*M*_2_.*j* = 1, …, *M*_1_. Since the spatial colored matrix, $N_{l}^{1}$, and $N_{l}^{2}$ are also satisfied with $\text{E}[{\left(N_{l}^{1}\right)}^{\text{H}},N_{l}^{2}]=0(l=1,2,\ldots ,L)$, the influence of spatial colored noise is eliminated in Equation (8), i.e., the cross-covariance tensor, 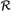
_21_, is not affected by the additive spatial colored noise. According to Equation (8), the relationship between the cross-correlation matrix $R_{21}=\frac{1}{L}{\left[Y_{2}\right]}_{\left(3\right)}^{\text{T}}{\left[Y_{1}\right]}_{\left(3\right)}^{*}$ and the cross-covariance tensor, 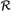
_21_, is shown in Equation (9).

(9) $$R_{21}=\left[\begin{array}{ccccccc} {\left[R_{21}\right]}_{1,1,1,1} & {\left[R_{21}\right]}_{1,1,1,2} & \cdots & {\left[R_{21}\right]}_{1,1,1,M_{1}} & {\left[R_{21}\right]}_{1,1,2,1} & \cdots & {\left[R_{21}\right]}_{1,1,N,M_{1}}\\ {\left[R_{21}\right]}_{1,2,1,1} & {\left[R_{21}\right]}_{1,2,1,2} & \cdots & {\left[R_{21}\right]}_{1,2,1,M_{1}} & {\left[R_{21}\right]}_{1,2,2,1} & \cdots & {\left[R_{21}\right]}_{1,2,N,M_{1}}\\ \vdots & \vdots & \vdots & \vdots & \vdots & \vdots & \vdots \\ {\left[R_{21}\right]}_{1,M_{2},1,1} & {\left[R_{21}\right]}_{1,M_{2},1,2} & \cdots & {\left[R_{21}\right]}_{1,M_{2},1,M_{1}} & {\left[R_{21}\right]}_{1,M_{2},2,1} & \cdots & {\left[R_{21}\right]}_{1,M_{2},N,M_{1}}\\ {\left[R_{21}\right]}_{2,1,1,1} & {\left[R_{21}\right]}_{2,1,1,2} & \cdots & {\left[R_{21}\right]}_{2,1,1,M_{1}} & {\left[R_{21}\right]}_{2,1,2,1} & \cdots & {\left[R_{21}\right]}_{2,1,N,M_{1}}\\ {\left[R_{21}\right]}_{2,2,1,1} & {\left[R_{21}\right]}_{2,2,1,2} & \cdots & {\left[R_{21}\right]}_{2,2,1,M_{1}} & {\left[R_{21}\right]}_{2,2,2,1} & \cdots & {\left[R_{21}\right]}_{2,2,N,M_{1}}\\ \vdots & \vdots & \vdots & \vdots & \vdots & \vdots & \vdots \\ {\left[R_{21}\right]}_{N,M_{2},1,1} & {\left[R_{21}\right]}_{N,M_{2},1,2} & \cdots & {\left[R_{21}\right]}_{N,M_{2},1,M_{1}} & {\left[R_{21}\right]}_{N,M_{2},2,1} & \cdots & {\left[R_{21}\right]}_{N,M_{2},N,M_{1}} \end{array}\right]$$

Then, the HOSVD [15,16] of the cross-covariance tensor, 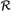
_21_, yields:

(10) $$R_{21}=S\times_{1}U_{1}\times_{2}U_{2}\times_{3}U_{3}\times_{4}U_{4}$$

where $S=R_{21}\times_{1}U_{1}^{H}\times_{2}U_{2}^{H}\times_{3}U_{3}^{H}\times_{4}U_{4}^{H}\in {\text{C}}^{N\times M_{2}\times N\times M_{1}}$ denotes the core tensor [15] satisfying the property of all-orthogonality, while **U**_1_, **U**_3_ ∈ ℂ*^N^*^×^*^N^*, **U**_2_ ∈ ℂ^*m*_2_^^×^^*m*_2_^ and **U**_4_ ∈ ℂ^*m*_1_^^×^^*m*_1_^ are unitary matrices. Since 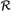
_21_ is a rank-*P* tensor, a cross-covariance subspace tensor, 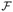
*_s_*, can be estimated by using the truncated HOSVD of 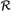
_21_, which can be written as:

(11) $$F_{s}=S_{s}\times_{1}U_{1s}\times_{2}U_{2s}\times_{3}U_{3s}\times_{4}U_{4s}$$

where **U***_is_*(*i* = 1, 2, 3, 4) contains the first *P* dominant singular vectors of **U***_i_*, $S_{s}=R_{21}\times_{1}U_{1s}^{\text{H}}\times_{2}U_{2s}^{\text{H}}\times_{3}U_{3s}^{\text{H}}\times_{4}U_{4s}^{\text{H}}$ as the reduced core tensor. Then substituting 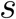
*_s_* into Equation (11), we have:

(12) $$F_{s}=R_{21}\times_{1}(U_{1s}U_{1s}^{\text{H}})\times_{2}(U_{2s}U_{2s}^{\text{H}})\times_{3}(U_{3s}U_{3s}^{\text{H}})\times_{4}(U_{4s}U_{4s}^{\text{H}})$$

According to the relationship between the cross-correlation matrix and its corresponding cross-covariance tensor in Equation (9) and the **Definition 3**, a new cross-correlation matrix, **R̄**_21_, is reconstructed from 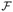
*_s_*, which can be expressed as:

(13) $${\bar{R}}_{21}=[(U_{1s}U_{1s}^{\text{H}})⊗(U_{2s}U_{2s}^{\text{H}})]R_{21}{\left[(U_{3s}U_{3s}^{\text{H}})⊗(U_{4s}U_{4s}^{\text{H}})\right]}^{*}$$

In the subspace method [14], the signal subspace matrix, **U***_s_*, is determined by using the truncated SVD of **R**_21_, i.e., **R**_21_ ≈ **U***_s_***Λ***_s_***V***_s_*. Inserting it into Equation (13), we have:

(14) $${\bar{R}}_{21}=\{[(U_{1s}U_{1s}^{\text{H}})⊗(U_{2s}U_{2s}^{\text{H}})]U_{s}\}\Lambda_{s}{\left\{{\left[(U_{3s}U_{3s}^{\text{H}})⊗(U_{4s}U_{4s}^{\text{H}})\right]}^{\text{T}}V_{s}\right\}}^{\text{H}}$$

According to Equation (14), using the truncated SVD of **R̄**_21_, the signal subspace, **Ū***_s_*, can be written as:

(15) $${\bar{U}}_{s}=[(U_{1s}U_{1s}^{\text{H}})⊗(U_{2s}U_{2s}^{\text{H}})]U_{s}$$

According to Equation (15), it is indicated that the signal subspace, **Ū***_s_*, and **U***_s_* span the same subspace. Hence, there exists a nonsingular matrix, **T** ∈ ℂ*^P^*^×^*^P^*, satisfied with **Ū***_s_* = (**A** ⊙ **B**)**T**. After obtaining the signal subspace, **Ū***_s_* the ESPRIT algorithm [7,8] is applied to estimate the DOD and DOA.

In order to estimate both the DOD and DOA, the signal subspace, **Ū***_s_* is divided into four submatrices: **Ū***_s_*_1_ = **Γ**_1_**Ū***_s_*, **Ū***_s_*_2_ = **Γ**_2_**Ū***_s_*, **Ū***_s_*_3_ = **Γ**_3_**Ū***_s_* and **Ū***_s_*_4_ = **Γ**_4_**Ū***_s_*, where $\Gamma_{1}=J_{\left(1\right)}^{\left(M_{2}-1\right)}⊗I_{N}$, $\Gamma_{2}=J_{\left(2\right)}^{\left(M_{2}-1\right)}⊗I_{N}$, $\Gamma_{3}=I_{M_{2}}⊗J_{\left(1\right)}^{\left(N-1\right)}$, $\Gamma_{4}=I_{M_{2}}⊗J_{\left(2\right)}^{\left(N-1\right)}$, $J_{\left(1\right)}^{\left(k\right)}=[I_{k}\;0_{k\times 1}]$, $J_{\left(2\right)}^{\left(k\right)}=[0_{k\times 1}\;I_{k}]$. In doing so, we have:

(16a) $$\Gamma_{2}{\bar{U}}_{s}=\Gamma_{1}{\bar{U}}_{s}\Psi_{t}$$

(16b) $$\Gamma_{4}{\bar{U}}_{s}=\Gamma_{3}{\bar{U}}_{s}\Psi_{r}$$

where **Ψ***_t_* = **T**^−1^**Φ***_t_***T**, **Ψ***_r_* = **T**^−1^ **Φ***_r_***T**, **Φ̄***_t_* = diag([*e^jπ^* ^sin^ ^*φ*_1_^, ⋯ , *e^jπ^* ^sin^ *^φ_P_^*]) and **Φ̄***_r_* = diag([*e^jπ^* ^sin^ ^*θ*_1_^, ⋯ , *e^jπ^* ^sin^ *^θ_P_^*]) contain the desired DOD and DOA information. Equation (16) can be solved by least squares (LS), then, **Ψ***_t_* and **Ψ***_r_* are obtained. Let **Φ̂***_t_* and **T̂** be the eigenvalue matrix and eigenvector matrix of **Ψ***_t_*. Then, the DOD of the *p*-th target is derived as:

(17) $$\begin{array}{cc} \varphi_{p}=\arcsin(\arg(\gamma_{p}^{t})/\pi ), & p=1,2,\ldots ,P \end{array}$$

where $\gamma_{p}^{t}$ is the *p*-th diagonal element of **Φ̄***_t_*. Note that **Ψ***_t_* and **Ψ***_r_* have the same eigenvector matrix, the diagonal matrix, **Φ̂***_r_*, can be determined as **Φ̂***_r_* = **T̂**^−1^**Ψ***_r_* **T̂**. Then, the diagonal elements of **Φ̂***_t_* and **Φ̂***_r_* in the same position correspond to the same target, i.e., the DOD and DOA are paired automatically. The DOA of the *p*-th target is derived as:

(18) $$\begin{array}{cc} \theta_{p}=\arcsin(\arg(\gamma_{p}^{r})/\pi ), & p=1,2,\ldots ,P \end{array}$$

where $\gamma_{p}^{r}$ is the *p*-th diagonal element of **Φ̂** *_r_*.

## 4. Computational Complexity Analysis and Remark

In order to analyze the computational complexity of the proposed method, it is necessary to know the complexity of the SVD algorithm. There are a lot of methods to compute SVD, and the computational complexities of them are different. In [17], it has been pointed out that orthogonal iteration is an efficient solution for SVD algorithm. The computational complexity of an *M* × *N* matrix truncated to rank *r* is *O*(*k_r_MNr*) by using this orthogonal iteration, where *k_r_* is a constant that depends on the design of the algorithm. The main computational burden of the proposed method, multi-SVD method, Chen's method and ESPRIT algorithm is the estimation of the signal subspace. In order to estimate the signal subspace, **Ū***_s_* the proposed method needs to calculate the truncated HOSVD of 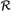
_21_ and the truncated SVD of **R̄**_21_. The truncated HOSVD of 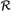
_21_ is equivalent to the truncated SVD of all its matrix unfolding, which needs *O*(*4k_r_M*_1_*M*_2_*N*^2^*P*). Additionally, the computational complexity of the truncated SVD of **R̄**_21_ is *O*(*k_r_M*_1_*M*_2_*N*^2^*P*). The total computational complexity of the proposed method is *O*(5*k_r_M*_1_*M*_2_*N*^2^*P*). In the multi-SVD method, the signal subspace is estimated by the truncated HOSVD of the third tensor, 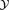. Thus, the computational complexity is *O*(3*k_r_MNLP*). Chen's method only needs the truncated SVD of **R**_21_ to estimate the signal subspace, which needs *O*(*k_r_M*_1_*M*_2_*N*^2^*P*). The ESPRIT algorithm uses the truncated SVD of the covariance matrix **R** = (1*/L*)**YY**^H^ to estimate the signal subspace, which needs *k_r_M*^2^*P.* According to the above analysis, the computational complexity of the proposed method is similar to the multi-SVD method, but higher than both Chen's method and the ESPRIT algorithm. However, the proposed method provides better angle estimation performance than all the aforementioned methods, which is demonstrated in the next section.

*Remark 1*: According to Equation (11), in order to obtain the signal subspace, **U***_is_*, of **U***_i_* (*i* = 1, 2, 3, 4), the necessary conditions for *M*_1_, *M*_2_, *N* and *L* are that: *M*_1_ ≥ *P*, *M*_2_ ≥ *P*, *N* ≥ *P, L* ≥ *P.* It is indicated that the maximum number of targets can be identified by the proposed method is min[*M*_1_,*M*_2_, *N, L*]. Thus, the number of targets that can be identified by the proposed method is smaller than ESPRIT algorithm. However, the proposed method performs well and provides better angle estimation performance in the presence of spatial colored noise, while the ESPRIT algorithm has marked performance degradation, especially in the low SNR region.

## 5. Simulation Results

In this section, some simulations are presented to evaluate the angle estimation performance of the proposed method in the presence of spatial colored noise. The multi-SVD algorithm [12], ESPRIT algorithm [10] and the method in [14] (denoted as Chen's method) are used to compare with the proposed method. We consider a MIMO radar system with *M* colocated antennas and *N* colocated antennas for the transmit and receive array, respectively. Both of the transmit array and receive array are half-wavelength spaced ULAs. *M* transmit antennas transmit *M* orthogonal waveforms, and the *m*-th transmitted waveform is the *m*-th row of **S** ∈ ℂ*^K^*^×^*^K^*, where $S=(1+j)/\sqrt{2}H_{K}$, and **H***_K_* is the *K* × *K* Hadamard matrix. The number of samples per pulse period is *K* = 256, and the pulse repetition interval is *T_r_* = 5 us. There exists three uncorrelated targets located at (*φ*_1_, *θ*_1_) = (30°, −30°), (*φ*_2_, *θ*_2_) = (−40°, 10°) and (*φ*_3_, *θ*_3_) = (10°, 10°), and the reflection coefficients of the targets are ${\left\{\beta_{p}\right\}}_{p=1}^{3}=1$. The Doppler shifts are ${\left\{f_{dp}\right\}}_{p=1}^{3}=\{300,400,500\}\text{Hz}$. The spatial colored noise is modeled as a spatial complex autoregressive (AR) model of second-order with the coefficients *z* = [1, −1,0.2] [12], and the root mean square error (RMSE) of the angle estimation is defined as:

(19) $$\text{RMSE}=\frac{1}{2Q}\sqrt{\sum_{i=1}^{Q}\left({\hat{\varphi }}_{i}-\varphi_{i})+({\hat{\theta }}_{i}-\theta_{i}\right)}$$

where *Q* is the number of Monte Carlo trials and *φ̂_i_* and *θ̂_i_* are the estimation of DOD *φ_i_* and DOA *θ_i_* of the *i*-th Monte Carlo trial.

**Figure 2.**
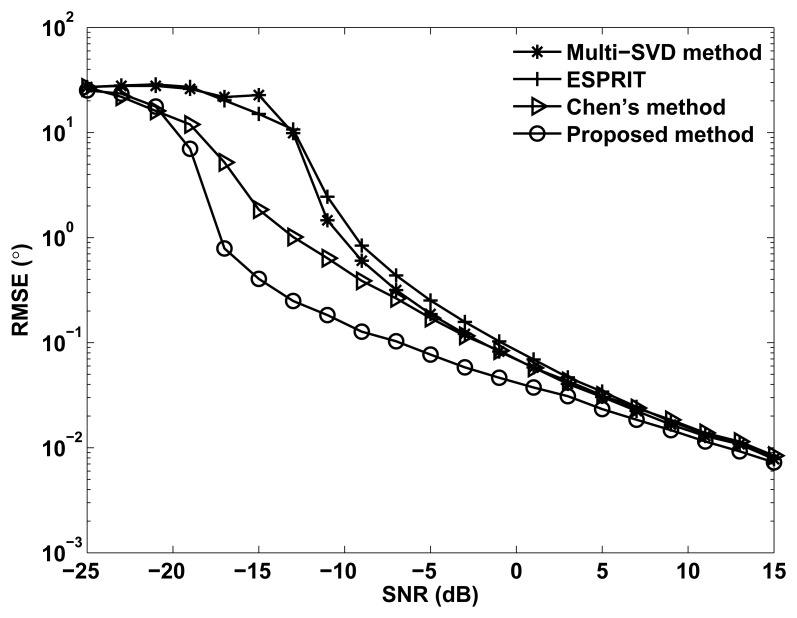
Root mean square error (RMSE) *versus* SNR for *P* = 3 targets.

Figure 2 shows the RMSE of angle estimation of different methods *versus* SNR, where *M* = *N* = 12, *M*_1_ = 3, *L* = 100 and *Q* = 200. It is shown in Figure 2 that the ESPRIT and multi-SVD algorithm provide worse angle estimation performance than Chen's method and the proposed method, especially at the low SNR region. This is because the ESPRIT and multi-SVD method cannot eliminate the influence of spatial colored noise. It also can be observed that Chen's method provides better angle estimation performance than the ESPRIT algorithm, which is consistent with [14]. Owing to taking the multidimensional structure into account and using the cross-covariance tensor technique, the proposed method can eliminate the spatial colored noise more efficiently. Thus, the proposed method outperforms all aforementioned methods, especially at the low SNR region.

**Figure 3.**
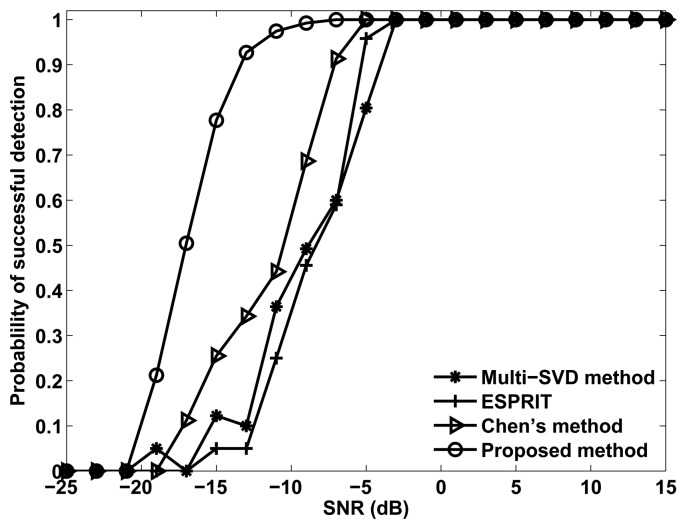
Probability of successful detection *versus* SNR.

Figure 3 shows the probability of the successful detection of the different methods *versus* SNR, where *M* = *N* = 12, *M*_1_ = 3, *L* = 100 and *Q* = 200. Successful detection requires that the absolute error of both DOD and DOA for all three targets are within 0.5°. It can be seen from Figure 3 that all the methods exhibit a 100% successful detection at high SNR values. As the SNR decreases, the probability of successful detection decreases for each method at a certain point, which is known as the SNR threshold. It also can be seen that the proposed method has a lower SNR threshold than Chen's method, ESPRIT and the multi-SVD method, owing to the super capability of eliminating the spatial colored noise.

**Figure 4.**
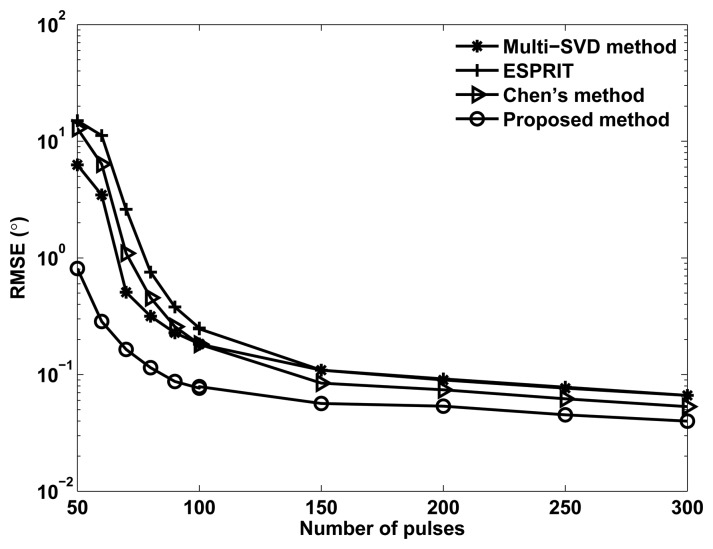
RMSE *versus* pulses for *P* = 3 targets.

Figure 4 shows the RMSE of angle estimation of different methods *versus* the number of pulses, where *M* = *N* = 12, *M*_1_ = 3, SNR= −5dB and *Q* = 200. It can be seen in Figure 4 that the angle estimation performance of all methods can be improved with pulse increases, and the proposed method provides better angle estimation performance than Chen's method, ESPRIT and the multi-SVD algorithm.

## 6. Conclusions

In this paper, a tensor-based subspace approach is presented to DOD and DOA estimation for bistatic multiple-input multiple-output (MIMO) radar in the presence of spatial colored noise. The proposed method exploits the the multidimensional structure inherent in the received signals to construct a third-order measurement tensor. Then, two sub-tensors are obtained from the measurement tensor, which can be used to formulate a cross-covariance tensor for eliminating the influence of spatial colored noise. Finally the DOD and DOA can be estimated in conjunction with the ESPRIT method. The proposed method has better angle estimation performance than Chen's method, ESPRIT and the multi-SVD method, especially at the low SNR region. Several simulation results have verified the performance of the proposed method.
